# Supplementary material for: Incorporation of Nanocatalysts for the Production of Bio-Oil from Staphylea holocarpa Wood
Source: Polymers (Basel). 2022 Oct 17;14(20):4385. doi: 10.3390/polym14204385 (PMC9609867; doi:10.3390/polym14204385)
Supplement: Supplementary file 1 [file polymers-14-04385-s001.zip › polymers-1947226-supplementary.pdf]

Supplementary Materials

Table S1. GC–MS analysis of *S. holocarpa* (methanol) sample.

No.	Retention Time (min)	Peak Area (%)	Component
1	2.72	1.02	Pentanoic acid, 2,2-dimethyl-, 1,2,3-propanetriyl ester
2	3.95	2.34	l-Alanine, N-methoxycarbonyl-, butyl ester
3	4.79	2.48	N,N-diethyl-Formamide
4	6.21	1.77	2-ethyl-1-Hexanol
5	7.25	2.65	Maltol
6	8.29	2.37	2,3-dihydro-3,5-dihydroxy-6-methyl-4H-Pyran-4-one
7	9.82	2.47	5-Hydroxymethylfurfural
8	10.05	1.64	Melezitose
9	11.25	3.05	1-(2-hydroxy-5-methylphenyl)-Ethanone
10	12.61	2.00	Decanedioic acid, 3,8-dioxo-, dimethyl ester
11	15.82	0.94	3,4,5-trimethoxy- Phenol
12	16.53	2.98	N-methyl-N-[4-(3-hydroxypyrrolidinyl)-2-butynyl]-Acetamide
13	17.66	5.50	(E)-4-(3-Hydroxyprop-1-en-1-yl)-2-methoxyphenol
14	19.59	1.56	(-)-Spathulenol
15	20.40	2.81	n-Hexadecanoic acid
16	20.77	0.97	3,5-Dimethoxy-4-hydroxycinnamaldehyde
17	20.88	1.37	1-Propyl-3,6-diazahomoadamantan-9-ol
18	22.43	4.00	12-Octadecadienoic acid (Z,Z)-9

Table S2. GC–MS analysis of *S. holocarpa* (benzene/ethanol) sample.

No.	Retention Time (min)	Peak Area (%)	Component
1	4.88	21.37	N,N-diethyl-Formamide
2	6.32	18.64	2-ethyl-1-Hexanol
3	9.74	1.18	4,5-dihydro-3-methyl-1-propyl-1H-Pyrazole
4	13.95	2.05	4-O-.beta.-D-galactopyranosyl-.beta.-D-Glucopyranose
5	17.61	1.11	1-methyl-N-vanillyl-(+)-2-Phenethanamine
6	19.29	1.34	(E)-2,6-Dimethoxy-4-(prop-1-en-1-yl)phenol
7	19.96	3.89	2-methyl- Hexadecanoic acid
8	20.38	1.04	Estra-1,3,5(10)-trien-17.beta.-ol

9	20.47	0.78	1,2-Benzenedicarboxylic acid, bis(2-methylpropyl) ester
10	22.05	2.41	11-Octadecenoic acid, methyl ester
11	22.33	3.29	Heptadecanoic acid, 15-methyl-, methyl ester
12	22.41	1.66	(Z)-18-Octadec-9-enolide

Table S3. GC–MS analysis of *S. holocarpa* (ethanol/methanol) sample.

No.	Retention Time (min)	Peak Area (%)	Component
1	4.09	2.21	Dihydroxyacetone
2	7.17	2.17	Clindamycin
3	8.26	4.36	2,3-dihydro-3,5-dihydroxy-6-methyl-4H-Pyran-4-one
4	9.74	3.59	5-Hydroxymethylfurfural
5	10.38	1.74	Melezitose
6	10.73	1.52	Melezitose
7	11.23	1.93	Thymol
8	13.53	2.35	d-Mannose
9	15.77	2.29	l-Gala-l-ido-octonic lactone
10	17.61	2.71	(E)-4-(3-Hydroxyprop-1-en-1-yl)-2-methoxyphenol
11	19.25	1.62	3-(4-hydroxy-3-methoxyphenyl)-2-Propenoic acid
12	19.96	5.56	Hexadecanoic acid, methyl ester
13	20.37	2.37	n-Hexadecanoic acid
14	22.04	4.01	(E)-9-Octadecenoic acid, methyl ester
15	22.32	6.00	Methyl stearate
16	22.40	2.50	1-Heptatriacotanol

Table S4. LC–QTOF–MSS analysis of *S. holocarpa* wood methanol extractives

No.	Retention Time (min)	Measured *m/z*	Component
1	8.60	438.17	Kraussianone 3
2	8.70	192.15	alpha-Ionone
3	8.70	82.08	Methylene cyclopentane
4	11.40	406.18	(E)-1-[2,4-Dihydroxy-3-(3-methyl-2-butenyl)phenyl]-3-(2,2-dimethyl-8-hydroxy-2H-benzopyran-6-yl)-2-p
5	12.00	278.15	Ditertbutyl phthalate
6	12.00	406.18	(E)-1-[2,4-Dihydroxy-3-(3-methyl-2-butenyl)phenyl]-3-(2,2-dimethyl-8-hydroxy-2H-benzopyran-6-yl)-2-p
7	12.00	576.36	Collettinside I

8	12.00	630.39	Macrophyllic acid
9	12.20	620.39	Cauloside B
10	12.30	296.14	Isocryptotanshinone
11	12.50	550.31	Cymarol
12	12.70	418.31	Maesaquinone
13	12.70	462.34	Trihydroxybufosterocholenic acid
14	12.70	506.36	13beta,17beta-Epoxyalisol A
15	12.70	796.49	Bupleuroside IX
16	12.80	114.10	beta-Heptenol
17	13.00	422.17	Eryvarin F
18	13.10	212.07	Antiarolaldehyde
19	13.10	272.09	Arbutin
20	13.10	268.13	7,8-Dihydroxy-isobutyryl-thymol
21	13.10	328.15	Rhododendrin
22	13.10	424.21	Didrovaltratum
23	13.10	440.18	Kushenol L
24	13.10	826.43	Dianoside C
25	13.10	184.07	Aucubigenin
26	14.10	286.23	5beta-Hydro-8,11,13-abietatrien-6alpha-ol
27	14.10	452.31	Dresigenin B
28	14.30	278.15	Ditertbutyl phthalate
29	14.30	148.02	Phthalic anhydride
30	14.90	766.48	Ilekudinoside I
31	15.00	272.09	Arbutin
32	15.00	424.21	Didrovaltratum
33	15.00	440.18	Kushenol L
34	15.00	634.41	16beta-Hydroxy-18beta-H-oleanolic acid-28-O-beta-D-glucopyranoside
35	15.00	184.07	Aucubigenin
36	15.40	460.32	12-Keto-porrigenin
37	15.50	310.16	7-(4''-Hydroxy-3''-methoxyphenyl)-1-phenyl-hept-4-en-3-one
38	15.50	504.34	Camelliagenin E
39	15.50	548.37	13beta,17beta-Epoxyalisol A 24-acetate
40	15.60	284.21	DELTA6-Dehydroferruginol
41	15.60	148.04	Allyl propyl disulfide
42	15.70	710.39	5-Ene-methyl-cholate-3-O-beta-D-glucuronopyranosyl-(14)-

			alpha-L- rhamnopyranoside
43	15.80	246.23	3-Phenyldodecane
44	15.90	598.31	Cinobufagin-3-hydrogen suberate
45	16.00	330.28	L-(-)-alpha-Monopalmitin
46	16.00	540.31	Resibufogenin 3-hydrogen suberate
47	16.00	148.04	Allyl propyl disulfide
48	16.00	128.12	3-Octanone
49	16.40	824.53	Dregeoside B
50	16.70	604.40	Hederagenin 3-O-arabinoside
51	16.70	114.10	beta-Heptenol
52	16.70	128.12	3-Octanone
53	16.80	428.29	Sisalagenone
54	16.80	516.34	Methyl 11-oxoasiatate
55	16.90	406.27	Ardisianone
56	17.00	170.13	Sobrerol
57	17.10	480.31	Commisterone
58	17.20	448.32	Isocarneagenin
59	17.30	480.27	3,4-Dihydro-excelsin
60	17.30	432.32	Chlorogenin
61	17.40	538.29	Euphorbia factor Ti2
62	17.40	618.41	Oleanolic acid-28-O-beta-D-glucopyranoside
63	17.60	530.36	25-O-Acetylcimigenol
64	17.60	768.50	Gypenoside XIV
65	17.70	394.21	Bixin
66	17.70	634.41	16beta-Hydroxy-18beta-H-oleanolic acid-28-O-beta-D-glucopyranoside
67	17.80	432.32	Chlorogenin
68	17.80	508.34	Lemmasterone
69	17.80	726.36	Nimbolidin E
70	17.90	700.26	(-)-Olivil-4',4''-di-O-beta-D-glucopyranoside
71	18.00	656.23	6-O-alpha-D-Galactopyranosylharpagoside
72	18.00	618.41	Oleanolic acid-28-O-beta-D-glucopyranoside
73	18.10	508.34	Lemmasterone
74	18.10	530.36	25-O-Acetylcimigenol
75	18.10	622.45	20(R)-Ginsenoside-Rh2
76	18.40	128.12	3-Octanone
77	18.70	442.34	Abrisapogenol J

78	19.00	248.18	(-)-Methyl selina-3,11-dien-14-oate
79	19.20	480.34	Brassinolide
80	19.60	466.36	Pentahydroxybufostane
81	19.60	482.34	26,27-Dihydroxy-lanosta-7,9(11),24-trien-3,16-dione
82	19.60	526.36	Tsugaric acid A
83	19.60	746.52	Azralidoside
84	19.80	466.36	Pentahydroxybufostane
85	20.30	272.25	gamma-Camphorene
86	20.80	713.54	Celebroside
87	20.90	572.41	22-O-Angeloyl theasapogenol B
88	20.90	614.47	Gardnerilin A
89	21.00	418.31	Maesaquinone
90	21.00	450.28	Celastrol
91	21.20	432.33	Chlorogenin
92	21.80	446.34	22E,24R-Ergosta-7,22-diene-3beta,5alpha,6beta,9alpha-tetraol
93	22.00	446.34	22E,24R-Ergosta-7,22-diene-3beta,5alpha,6beta,9alpha-tetraol
94	22.10	428.37	21-Hydroxy-30-norhopan-22-one
95	22.20	408.38	Neohopadiene
96	23.40	656.30	Dicliripariside A
97	23.70	656.30	Dicliripariside A
98	23.70	614.36	Toxiferine I
99	24.50	624.49	Asitrilobin A
100	24.80	580.47	Corossoline
101	24.90	624.49	Asitrilobin A
102	25.20	580.47	Corossoline
103	26.00	582.48	Gardnerilin B
104	27.00	608.50	Uvarigrin
105	27.10	434.23	Glabcensin Y
106	27.10	478.26	2-Deacetyldecinnamoyltaxinine E
107	27.10	568.31	Adouetine Y
108	27.20	131.09	Alloisoleucine
109	27.20	163.12	Acetylcholine
110	27.30	301.24	(E,E,E)-N-(2-Methylpropyl)-hexadeca-2,6,8-trien-10-ynamide
111	27.40	315.26	Holadysine

Table S5. Py/GC–MS analysis of *S. holocarpa* wood.

No.	Retention Time (min)	Peak Area (%)	Component
1	1.54	24.1	Dimethyl ether
2	1.59	0.42	nitroso- Methane
3	1.64	4.19	1-Methylcyclopropene
4	1.83	0.54	1,3-Pentadiene
5	1.94	2.65	1,3-Cyclopentadiene
6	2.01	0.91	Methylenecyclopropane
7	2.12	0.22	1,3-Butadiene
8	2.30	0.21	Cyclopropylacetylene
9	2.61	0.12	1-methyl-1,3-Cyclopentadiene
10	2.65	0.2	1-methyl-1,3-Cyclopentadiene
11	2.85	8.96	Benzene
12	3.23	2.17	Benzene
13	3.34	0.97	Benzene
14	5.07	3.17	Toluene
15	5.43	0.47	Toluene
16	5.56	0.15	N-Benzyl-N'-(1-benzylamino-2,2,2-trichloroethyl)-p-tolylcar boxamidine
17	7.87	0.15	Ethylbenzene
18	8.12	0.28	Benzene, 1,3-dimethyl-
19	8.73	0.23	1,3,5,7-Cyclooctatetraene
20	8.88	0.28	1,3,5,7-Cyclooctatetraene
21	12.06	1.45	Indene
22	12.24	0.21	Benzaldehyde, 2-nitro-, diaminomethylidenhydrazone
23	13.93	2.63	6-(methylsulfonyl)- 1H-Benzimidazol-2-amine
24	14.04	0.21	1-methyl-1,2-propadienyl- Benzene
25	14.14	0.52	Naphthalene
26	14.62	2.43	Naphthalene
27	14.91	0.03	2-Naphthalenol, 1,2-dihydro-, acetate
28	16.37	0.3	2-methyl- Naphthalene
29	16.55	0.38	1-methyl- Naphthalene
30	18.53	0.76	Biphenylene
31	18.74	0.07	4,7-dimethyl-1,3-Isobenzofurandione
32	21.97	0.35	9-Ethynyl-9-fluorenol
33	22.64	0.33	Phenanthrene

34	23.35	0.96	11-Methylnonacosane
35	23.77	0.19	1-(3-(Uracil-1-yl)propyl)-6-azathymine
36	24.01	0.11	4H-Cyclopenta[def]phenanthrene
37	24.09	0.19	1,1'-(1,2-propadienylidene)bis- Benzene
38	24.23	0.03	2-Nonenoic acid, 2-methyl-, ethyl ester
39	24.81	0.39	1-Aza-4-phosphacyclohexane, 6-dimethyl-4-oxo-4-phenyl-1-(2-hydroxyethyl)-2
40	25.51	0.24	Fluoranthene
41	25.76	0.18	Pyrene
42	25.93	0.07	Diphenyl sulfoxide
43	26.03	0.43	Pyrene
44	26.82	0.04	2,5-Dimethylbenzselenazole
45	26.91	0.14	11H-Benzo[b]fluorene
46	27.10	0.14	11H-Benzo[b]fluorene
47	27.27	0.13	Naphtho[2,1-d][1,3]dioxepin, 8,8,11a-tetramethyl-, [5aR-(5a.alpha.,7a.beta.,11a.alpha.,11b.beta.)]-dodecahydro-5a
48	27.39	0.05	Acetic acid, 2-acetoxymethyl-6-(2,4-dioxo-3,4-dihydro-2H-pyrimidin-1-yl)-tetrahydropyran-3-yl ester
49	28.43	0.18	4-chloro-9(10H)-Anthracenone
50	28.79	0.04	Fumaric acid, 2-chloroethyl heptadecyl ester
51	29.00	0.41	Benzo[ghi]fluoranthene
52	29.11	0.18	Benzo[c]phenanthrene
53	29.40	0.25	5-Bromo-1-isopropyl-6-hydroxypyridazin-6-one
54	29.55	0.08	1-[5-fluoropentyl]-4-[(3,4-dichlorophenyl)acetyl]- Piperazine
55	29.62	0.14	1,6,7,8,9,11a,12,13,14,14a-decahydro-1,13-dihydroxy-6-methyl-,[1R-(1R*,2E,6S*,10E,11aS*,13S*,14aR*)]-4H-Cyclopent[f]oxacyclotridecin-4-one
56	29.80	0.03	Sclaral (sclareolide lactol)
57	29.85	0.12	Nonanenitrile
58	29.96	0.08	1-bromo-1-(3-methyl-1-pentenylidene)-2,2,3,3-tetramethyl-Cyclopropane
59	30.01	0.35	tris(.eta.3-2-propenyl)-Iron
60	30.18	0.31	1-Phenyl-3,5,7-trimethyl-6,7(8H)-dihydropyrazolo(3,4-b)(1,4)diazepine
61	30.32	0.32	5,5-Diethylpentadecane
62	30.36	0.26	octahydro-4a-hydroxy-5-methyl-4-nitro-1(2H)-Naphthaleno

			ne
63	30.47	0.3	E-10-Methyl-11-tetradecen-1-ol propionate
64	30.52	0.36	Fumaric acid, 4-bromophenyl nonyl ester
65	30.62	0.31	9-Ethyl-1-nitrocarbazole
66	30.74	0.58	Succinic acid, 2-methylphenyl octadecyl ester
67	30.82	0.25	Dichloroacetic acid, undecyl ester
68	30.86	0.19	Fumaric acid, nonyl tetradec-3-enyl ester
69	30.89	0.23	Cyclododecanecarboxylic acid
70	31.00	1.06	1,1,4,4-tetrachloro-1,3-Butadiene
71	31.09	0.33	1-(2-Hydroperfluoroisobutyl)-3,3-dimethyldiaziridine
72	31.15	0.5	cis-5-Hydroxy-cis-3,trans-5-dimethylcyclohexane-1,cis-2-dica rboxylic acid
73	31.28	1.54	1-Docosanethiol
74	31.36	0.22	3-octyl-, cis- Oxiraneoctanoic acid
75	31.38	0.08	2-(2-t-Butyl-4-oxotetrahydrocyclopenta[1,3]dioxin-4a-ylmeth yl)acrylic acid, butyl ester
76	31.40	0.16	2,4-dimethyl-9-Oxadodecan-4-ol
77	31.51	2.05	Benzo[j]fluoranthene
78	31.77	1.73	Heptadecafluorononanoic acid, octadecyl ester
79	31.83	0.47	1,2,3,4-tetrahydro-1-(1-oxopropyl)-5-(2-piperidinyl)-Pyridine
80	31.89	1.65	Phosphetane, 1-bromo-2,2,3,4,4-pentamethyl-, 1-sulfide
81	32.09	1.59	methyl-2,3-O-(ethylboranediyl)- .beta.-d-Mannofuranoside
82	32.27	0.61	5-Methoxy-4,4,6-trimethyl-7-oxabicyclo[4.1.0]heptan-2-one
83	32.34	0.5	Trichloroacetic acid, 4-hexadecyl ester
84	32.52	1.37	Oleyl oleate
85	32.59	1.03	Distearyl thiodipropionate
86	32.71	0.71	(Z)-9-Hexadecenoic acid, octadecyl ester
87	32.82	0.54	Diethylmalonic acid, monochloride, dodec-9-ynyl ester
88	32.89	0.58	2-Hydroxy-1,1,10-trimethyl-6,9-epidioxydecalin
89	32.99	0.36	2-bromo- Hexadecanoic acid
90	33.06	0.49	Dimethylmalonic acid, dodecyl pentachlorophenyl ester
91	33.14	1.18	(1-butylhexadecyl)- Cyclohexane
92	33.27	0.45	1,5-dimethyl-7-Oxabicyclo[4.1.0]heptane
93	33.36	0.35	Dasycarpidan-1-methanol, acetate (ester)
94	33.42	0.26	Dasycarpidan-1-methanol, acetate (ester)
95	33.45	0.97	5-Methyl-Z-5-docosene
96	33.67	0.58	7-Octadecanone

97	33.71	0.33	1,1,1,4,4-Pentamethyl-4-vinyldisilethylene
98	33.77	0.2	5,6,7,8-tetrahydro-1,3,6,7,8-pentamethyl-2,4(1H,3H)-Pteridinedione
99	33.87	0.52	3,9-diethyl-,2,4,8,10-tetraoxa-3,9-dibora-3,9-Dispiro[5,5]undecane
100	33.91	0.19	6,7-difluoro-2-(hexahydro-1H-1,4-diazepin-1-yl)-1,3-Benzoxazole
101	33.98	0.27	1-N-(4-fluorophenyl)-4-methyl-1-Piperazinepropanamide
102	34.03	0.2	5-Methoxy-cyclooctene
103	34.08	0.33	7-Ethyl-4-tetradecen-6-one
104	34.23	0.7	Acetic acid, chloro-, octadecyl ester
105	34.29	0.2	2-Acetylamino-3-cyano-propionic acid
106	34.33	0.42	Geranyl isovalerate
107	34.43	0.44	3-(2,5-dihydro-4,5,5-trimethyl-2-pyrimidinyl)-3-methyl-, (.+/-.)-2-Butanone
108	34.52	0.78	erythro-7,8-Bromochlorodisparlure
109	34.63	0.74	Dichloroacetic acid, undec-2-enyl ester
110	34.76	0.5	4,4',4''-borylidynetris- Morpholine
111	34.82	0.47	6-cyclohexyl- Undecane
112	34.89	0.64	1-Pentatriacontanol
113	35.03	0.45	N-(4-fluorophenyl)-4-methyl-1-Piperazinepropanamide
114	35.07	0.25	Heneicosane, 11-cyclopentyl-
115	35.17	0.33	6-Tetradecanesulfonic acid, butyl ester
116	35.27	0.12	2-Amino-8-[3-d-ribofuranosyl]imidazo[1,2-a]-s-triazin-4-one
117	35.45	0.39	2,2,6-trimethyl-, trans-1-Oxaspiro[2.5]octan-4-one
118	35.47	0.26	(1-decylundecyl)- Cyclohexane
119	35.55	0.23	1-Heneicosanol
120	35.59	0.43	2-Methyloctadeca-7,8-diol bis(trifluoroacetate)
121	35.66	0.47	7-Ethyl-4-tetradecen-6-one
122	35.81	0.25	6-cyclohexyl-Dodecane
123	35.87	0.1	1-Hentetracontanol
124	35.94	0.06	3-cyclohexyl-Decane

Table S6. Py/GC–MS analysis of *S. holocarpa* wood/NiO.

No.	Retention Time (min)	Peak Area (%)	Component
1	1.26	0.10	2,6-Dimethyl-8-oxoocta-2,6-dienoic acid, methyl ester
2	1.54	15.63	Ethanol

3	1.64	4.90	1-Methylcyclopropene
4	1.83	0.62	(E)- 1,3-Pentadiene
5	1.88	0.37	1,2-Dimethyl cyclopropene
6	1.94	1.52	1,3-Cyclopentadiene
7	1.99	0.86	Bicyclo[2.2.1]hept-5-ene-2-carbonitrile
8	2.10	0.29	1,3-Butadiene
9	2.60	0.24	(Z)-1,3,5-Hexatriene
10	2.65	0.10	(Z)-1,3,5-Hexatriene
11	2.85	6.33	Benzene
12	3.32	0.82	Benzene
13	3.51	0.35	Trimethyl(2,2,2-trifluoroethoxy)silane
14	5.07	1.72	Toluene
15	5.50	0.20	Toluene
16	7.87	0.16	o-Xylene
17	8.17	0.35	p-Xylene
18	8.69	3.35	o-Xylene
19	11.02	1.16	Mesitylene
20	12.08	4.85	Indene
21	14.68	3.39	Naphthalene
22	16.40	0.25	2-methyl-Naphthalene
23	16.58	2.47	1-methyl-Naphthalene
24	18.55	1.11	Biphenylene
25	19.21	0.13	.alpha.-methyl-.alpha.-propyl- Benzenemethanol
26	19.42	0.04	5-Benzofuranacetic acid, 6-ethenyl-2,4,5,6,7,7a-hexahydro-7a-hydroxy-3,6-dimethyl-.alpha.-methylene-2-oxo-, methyl ester
27	22.82	2.47	Anthracene
28	25.58	0.40	Fluoranthene
29	26.04	0.27	Pyrene
30	26.68	0.09	1,2-Cyclopentanedicarboxylic acid, 4-[(trimethylsilyl)methylene]-, dimethyl ester, trans-
31	26.93	0.16	11H-Benzo[b]fluorene
32	27.97	0.65	Octadecanenitrile
33	28.91	0.06	Taraxasterol
34	29.03	1.01	Benzo[c]phenanthrene
35	29.56	0.23	2,6-diamino-5-(2-furfurylidenamino)- Pyrimidin-4-ol
36	29.75	0.64	2-Quinoxalineacetic acid,

			1-[(ethylamino)carbonyl]decahydro-3-oxo-, ethyl ester
37	29.87	0.31	N-(1-adamantyl)- 1-Pyrrolin-2-amine
38	29.96	0.24	5-Bromo-1-isopropyl-6-hydroxypyridazin-6-one
39	30.00	0.67	S-((3-(2,6-Dichlorophenyl)-5-methyl-4-isoxazolyl)methyl) O,O-dimethyl dithiophosphate
40	30.17	0.18	3-hydroxy-21-methoxy-20-oxo-, methyl ester, (3.beta.)- 30-Norlupan-28-oic acid
41	30.19	0.20	1,3-dimethyl-2,4,6-triphenyl- Piperidin-4-ol
42	30.23	1.57	Fumaric acid, 4-cyanophenyl decyl ester
43	30.63	1.27	1-Chloro-1-n-decyloxy-1-silacyclopentane
44	30.72	1.04	Hydroquinone bis(trimethylsilyl) ether
45	30.83	0.52	16-Hentriacontanone
46	30.88	1.15	8-propoxy-Cedrane
47	31.03	0.53	Octadecanoic acid, 12-oxo-, methyl ester
48	31.12	4.15	Fumaric acid, 2-methylallyl undecyl ester
49	31.50	2.12	Perylene
50	31.64	0.67	(Z)- 7-Hexadecenal
51	31.76	1.33	pentachloro- Benzene
52	31.95	1.99	Fumaric acid, 2-chloroethyl dodecyl ester
53	32.02	0.94	Methyl tetrahydroionol
54	32.19	1.72	2,6,10,14-Tetramethyl-7-(3-methylpent-4-enylidene) pentadecane
55	32.31	1.88	Epi-inositol tri-butaneboronate
56	32.43	0.72	(4-Methoxy-6-morpholin-4-yl-[1,3,5]triazin-2-yloxy)acetonitr ile
57	32.49	0.63	Bacchotricuneatin
58	32.56	0.60	.beta. Carotene
59	32.61	0.96	13-Methyl-Z-14-nonacosene
60	32.90	2.49	4,6-bis(1,1-dimethylethyl)-3-nitro-3,5-Cyclohexadiene-1,2-dio ne
61	33.03	1.25	p-Nitrophenyl nonyl ether
62	33.08	0.77	1a,2,5,5a,6,9,10,10a-octahydro-5,5a-dihydroxy-4-(hydroxyme thyl)-1,1,7,9-tetramethyl-11-oxo-1H-2,8a-methanocyclopenta[a]cyclopropa[e]cyclodecen-6-yl ester, [1aR-(1a.alpha.,2.alpha.,5.beta.,5a.beta.,6.beta.,8a.alpha.,9.alp ha.,10a.alpha.)]- Hexadecanoic acid
63	33.28	2.08	1-Ethyl-3,trans-(1,1-dimethylethyl)-4,cis-methoxycyclohexan -1-ol

64	33.63	2.07	5-amino-3-butyl-4-propyl- Isoxazole
65	33.76	0.81	8-(3-ethoxypropylamino)-1,3-dimethyl-3,9-dihydro-Purine-2,6-dione
66	33.80	0.27	Endothal dimethyl ester
67	33.84	0.20	12-Methyl-E,E-2,13-octadecadien-1-ol
68	33.90	0.32	6,10-dimethyl-9-Undecen-2-one
69	34.08	1.32	Fumaric acid, decyl propargyl ester
70	34.39	1.65	Octadecanoic acid, 16-oxo-, methyl ester
71	34.51	0.57	7-(5-acetylaminomethylthien-2-yl)- Heptanoic acid
72	34.82	1.12	1-(1-Methoxycyclopropyl)-3-methylbut-2-en-1-ol
73	34.83	0.07	Hexadecanenitrile
74	34.87	0.18	2-ethyl-2,7-Diazaspiro[4.4]nonane
75	35.00	0.41	Desipramine
76	35.12	0.32	2-cyclohexylimino-7a-methyl- Octahydro-benzo[b]furan
77	35.32	0.59	Butyl 14-methylhexadecanoate
78	35.43	0.26	N-acetyl- Isotomatidine
79	35.49	0.12	Isophthalic acid, cis-tetradec-3-enyl isohexyl ester
80	35.79	0.42	5-hydroxy-2,6,6-trimethyl-, [R*,R*-(E)]- 3-Heptenoic acid

Table S7. Py/GC–MS analysis of *S. holocarpa* wood/Co_3O_4.

No.	Retention Time (min)	Peak Area (%)	Component
1	1.55	19.21	Oxalic acid
2	1.59	0.41	nitroso- Methane
3	1.64	3.34	1,3-Butadiene
4	1.83	0.47	(E)-1,3-Pentadiene
5	1.95	2.65	1,3-Cyclopentadiene
6	2.61	0.54	1-methyl-1,3-Cyclopentadiene
7	2.65	0.17	5-methyl-1,3-Cyclopentadiene
8	2.86	6.07	Benzene
9	3.20	0.14	4-Cycloheptylpiperazinyl-1-carbothiohydrazide, 2-[1-(2-pyridyl)ethylidene]
10	3.29	0.84	Benzene
11	3.45	0.53	1,5-Hexadiyne
12	3.71	0.07	(E)-1-(5-Nitro-2-furyl)-2-bromo-2-methylethene
13	5.07	2.04	Toluene
14	5.47	0.82	Toluene
15	7.87	0.12	o-Xylene

16	8.12	0.53	1,3-dimethyl- Benzene
17	8.70	3.17	o-Xylene
18	12.08	0.59	Indene
19	14.68	4.19	Azulene
20	16.35	1.53	6-benzyl-3.alpha.-hydroxy-6-Azacholest-4-en-7-one
21	16.41	0.17	Spiro(9-methylenetricyclo[6.2.1.0(2,7)]undeca-2,4,6-triene)-1 1,1'-cyclopropane
22	16.57	1.31	2-methyl- Naphthalene
23	18.55	0.44	Biphenylene
24	22.71	3.09	Dibenzo[b,e]7,8-diazabicyclo[2.2.2]octa-2,5-diene
25	24.03	0.91	4H-Cyclopenta[def]phenanthrene
26	25.76	0.10	Pyrene
27	27.02	0.30	4-Decenoic acid, ethyl ester
28	27.87	0.03	1,10-Decanedioic acid, bis(DMOX) derivative
29	27.91	0.08	12'-hydroxy-2'-methyl-5'-(2-methylpropyl)-, (5'.alpha.)-Ergotaman-3',6',18-trione
30	28.05	0.60	Propiolic acid, 3-(1-hydroxy-2-isopropyl-5-methylcyclohexyl)-, ethyl ester
31	28.50	0.32	1,1,3,6-tetramethyl-2-(3,6,10,13,14-pentamethyl-3-ethyl-pent adecyl)cyclohexane
32	28.90	0.59	Octanoic acid (5-azepan-1-yl-furan-2-ylmethylene)-hydrazide
33	29.00	0.14	2-Isopropyl-4-trimethylsilylethynyloxazolidine-3-carboxylic acid, methyl ester
34	29.03	0.39	4-Methyl-2,6,7-trioxa-1-phosphabicyclo[2.2.2]octane 1-selenide
35	29.23	1.16	Hexatriacontyl pentafluoropropionate
36	29.41	0.40	O,N,N-triacetyl- Cyclohexan-1-ol-2-amine
37	29.68	1.66	1,1,3,6-tetramethyl-2-(3,6,10,13,14-pentamethyl-3-ethyl-pent adecyl)cyclohexane
38	29.83	1.56	dimethyl(2,2,2-trichloroethoxy)undecyloxy- Silane
39	30.05	1.53	i-Propyl 13-methyltetradecanoate
40	30.24	0.87	Norethandrolone

41	30.30	0.67	7-Methyl-1,5-diazacyclotetradecane
42	30.40	0.69	11,15-Dimethylhentriacontane
43	30.50	1.02	E,Z-2,15-Octadecadien-1-ol acetate
44	30.61	1.11	[1,1'-Bicyclohexyl]-4-carboxylic acid, 4'-butyl-, 4-pentylcyclohexyl ester
45	30.72	0.97	4-[[(2-methoxy-4-octadecenyl)oxy]methyl]-2,2-dimethyl-1,3-Dioxolane
46	30.84	0.59	tert-Hexadecanethiol
47	30.89	0.90	Methyl 11-eicosenoate
48	31.03	0.86	erythro-7,8-Bromochlorodisparlure
49	31.11	0.80	2,6-diethyl- Cyclohexanone
50	31.21	0.99	Ethanol, 2-butoxy-, phosphate (3:1)
51	31.35	2.45	2- Bromopropionic acid, octadecyl ester
52	31.58	1.83	2,3-dihydro-2-hydroxymethyl-5,7-dimethyl-Oxazolo[3,2-E]xanthine
53	31.61	0.65	2-[4-Methoxyphenyl-(1-methylazepan-2-ylidenamino)methylene]malononitrile
54	31.71	2.06	Cimetidine
55	31.89	1.50	1,4,4-trimethyl-2-oxo-, (1.alpha.,5.alpha.,6.alpha.)-(.+-.)-Bicyclo[3.1.0]hexane-6-carboxylic acid
56	32.03	1.04	2-Bromopropionic acid, 2-ethylcyclohexyl ester
57	32.16	1.40	trans-9,10-Epoxypentacosane
58	32.29	0.57	2-Octyldecyl butyrate
59	32.34	0.14	2,3-Anhydro-d-mannosan
60	32.39	0.63	3,7,11,15-Tetramethyl-hexadecanol, trimethylsilyl ether
61	32.49	0.75	9-Octadecenoic acid (Z)-, 2-(octadecyloxy)ethyl ester
62	32.59	1.16	5-Methyl-Z-5-docosene
63	32.65	0.48	Di-n-decylsulfone
64	32.88	2.22	Ethyl trans-4-decenoate
65	32.97	0.60	Ethyl 9-tetradecenoate
66	33.08	0.76	3,3,6-Trimethyl-1,4-heptadien-6-ol
67	33.16	0.59	.beta.-Methylether of 11-epi-dihydroartemisinin

68	33.27	0.65	1-[2-Deoxy-.beta.-d-erythro-pentofuranosyl]pyrrole-2,4-dicarboxamide
69	33.40	0.78	6,10,13-Trimethyltetradecanol
70	33.62	1.38	Ethyl 2-aza-3-oxatricyclo[3.3.3.0(1,5)]undecane-4-carboxylate
71	33.69	0.56	Imidazolo[1,5-a]pyrimidine-8-carboxylic acid, 1,2,3,5,6,7-hexahydro-2,5,5-trimethyl-1,3,7-trioxo-, ethyl ester
72	33.87	1.12	(S)-4-Piperidinecarboxylic acid, 1-(trifluoroacetyl)-, 1-methylpropyl ester
73	34.00	0.43	endo-Bicyclo[2.2.1]heptan-2-one, 5-(acetyloxy)-4,6,6-trimethyl-
74	34.13	0.49	Cyclononanone, oxime
75	34.17	0.61	5-Bromo-8-(2-hydroxy-1-naphthylmethyleneamino)quinoline
76	34.31	0.18	Pentafluoropropionic acid, tetradecyl ester
77	34.41	0.41	14-Methyldotriacontane
78	34.47	0.83	Perhydro-htx-2-one, 2-depentyl-, acetate ester
79	34.81	0.60	3,3-dimethyl-Bicyclo[2.2.1]heptane-2-thione
80	34.90	0.15	i-Propyl 23-methyl-tetracos-5,9-dienoate
81	34.97	0.21	bis-Octadecane, 1,1'-[1,3-propanediylbis(oxy)]
82	35.07	0.21	Undecanenitrile
83	35.11	0.10	2,6,10,14-Tetramethyl-7-(3-methylpent-4-enylidene) pentadecane
84	35.16	0.13	1R,3-cis-Diethoxy-5-trans-methylcyclohexane
85	35.23	0.14	3-Nonanol, 1,2;6,7-diepoxy-3,7-dimethyl-, acetate
86	35.30	0.12	6-diol, 2,6-dimethyl-2,7-Octadiene-1
87	35.34	0.06	5-Methyl-Z-5-docosene
88	35.63	0.28	1-Methyl-4-nitro-5-[(1,2-dimethyl-3-hydroxybutyl)amino]-(1H)-imidazole
89	35.65	0.04	2-pentyl-2-Nonenal

90	35.75	0.02	1,1,3,3-Tetraallyl-1,3-disilacyclobutane

Table S8. Py/GC−MS analysis of *S. holocarpa* wood/NiO+Co_3O_4.

No.	Retention Time (min)	Peak Area (%)	Component
1	1.55	25.51	Formic acid
2	1.59	0.74	nitroso-Methane
3	1.64	4.43	1-Methylcyclopropene
4	1.84	0.58	(Z)-1,3-Pentadiene
5	1.95	1.60	1,3-Cyclopentadiene
6	2.00	0.31	1,3-Cyclopentadiene
7	2.04	0.71	4-Pentyn-2-ol
8	2.15	0.24	Methylenecyclopropane
9	2.43	0.18	Cyclopropylacetylene
10	2.61	0.12	1-methyl-1,3-Cyclopentadiene
11	2.66	0.17	1-methyl-1,3-Cyclopentadiene
12	2.86	8.10	Benzene
13	3.16	0.00	1-Hexen-3-yne
14	3.37	0.69	Benzene
15	3.48	0.40	Benzene
16	5.07	3.01	Toluene
17	5.54	0.26	Toluene
18	7.87	0.14	Ethylbenzene
19	8.12	0.43	1,3-dimethyl-Benzene
20	8.71	1.23	5-methyl-1,6-Heptadien-3-yne
21	11.02	1.52	(1-methylethyl)-Benzene
22	11.06	0.51	2-propenyl-Benzene
23	12.06	1.52	Indene
24	13.93	2.42	2-ethoxy-Benzenamine
25	14.04	0.38	1-butynyl-Benzene
26	14.62	2.56	Naphthalene
27	14.89	0.11	Tricyclo[4.4.0.0(3,8)]dec-9-en-4-ol
28	14.94	0.10	[4.2.2]Propella-2,4,7,9-tetraene
29	15.06	0.19	2-(2-hydroxy-1-naphthylmethylamino)-Acetic acid
30	16.38	0.46	1,4-dihydro-1,4-Methanonaphthalene
31	16.56	0.55	2-methyl-Naphthalene

32	18.17	0.43	2-Phenyl-1,3-cyclohexadiene
33	18.28	0.06	1-phenyl-1-Penten-4-yn-3-ol
34	18.52	0.55	Biphenylene
35	20.25	0.10	Fluorene
36	22.62	1.27	Phenanthrene
37	25.18	0.13	1,8-di-1-propynyl-Naphthalene
38	25.52	0.17	Fluoranthene
39	25.77	0.07	Pyrene
40	26.03	0.29	Pyrene
41	26.12	0.02	Methdilazine
42	26.79	0.02	1-Phenanthrylene oxide
43	26.88	0.12	11H-Benzo[b]fluorene
44	27.07	0.17	11H-Benzo[b]fluorene
45	27.17	0.03	Cyclohexanecarboxylic acid, 4-butyl-, 4-butylphenyl ester
46	27.29	0.12	11H-Benzo[b]fluorene
47	28.19	0.14	(Z,Z,Z)-9,12,15-Octadecatrienoic acid, 2,3-bis[(trimethylsilyl)oxy]propyl ester,
48	28.23	0.02	1-methyl-4-acetylmethyl-4-[3-methoxyphenyl]- Piperidine
49	28.50	0.14	Benzo[ghi]fluoranthene
50	29.01	0.34	Benzo[ghi]fluoranthene
51	29.09	0.27	Naphthacene
52	29.36	0.80	3-(5-Hydroxy-2,2,6-trimethyl-7-oxa-bicyclo[4.1.0]hept-1-yl)-acrylic acid, methyl ester
53	29.69	0.37	9a-Methano-1-benzoxepin, octahydro-2,2,5a,9-tetramethyl-, [3R-(3.alpha.,5a.alpha.,9.alpha.,9a.alpha.)]- 2H-3
54	29.89	0.10	(3.beta.,5.alpha.,6.beta.,12.beta.)-Ergostane-3,5,6,12,25-pentol, 25-acetate
55	30.01	0.50	2-(4-fluorophenyl)-6-methyl-N-(1,1,3,3-tetramethylbutyl)- Imidazo[1,2-a]pyridin-3-amine
56	30.18	0.48	2-chloromethyl- Bicyclo[2.2.2]octan-1-ol

57	30.20	0.26	4,4,6-Trimethyl-6-phenyltetrahydro-1,3-oxazine-2-thione
58	30.28	0.60	dimethyl(2,2,2-trichloroethoxy)undecyloxy- Silane
59	30.40	0.35	1-Di(tert-butyl)silyloxydecane
60	30.54	0.43	6-Octadecenoic acid
61	30.57	0.70	Spiro[cyclohexanol-5,6'-piperidin-2'-one],6-butyl-, tetrahydropyran-6-yl ether
62	30.70	0.31	1H-1,2,3-Triazole-4-carboxylic acid, 1-(4-amino-1,2,5-oxadiazol-3-yl)-5-ethyl-, ethyl ester
63	30.74	0.26	Fumaric acid, hexyl 5-methoxy-3-methylpentyl ester
64	30.80	0.22	1-Decanol, 9-[(trimethylsilyl)oxy]-, trifluoroacetate
65	30.85	1.11	1-(hexadecyloxy)- 2-Propanol
66	31.00	0.52	Disparlure
67	31.07	0.34	16-Hydroxyhexadecanoic acid
68	31.11	0.49	trans-4,6,6-Trimethyl-7-oxo-8-oxabicyclo(2.2.2)octane-2-carboxylic acid
69	31.18	0.88	1-Cyclohexyl-4-pyrrol1-[1-(2-methoxyethyl)-1H-tetrazol-5-yl]propylmorphopiperazine
70	31.27	1.48	12'-hydroxy-2'-methyl-5'-(2-methylpropyl)-, (5'.alpha.)- Ergotaman-3',6',18-trione
71	31.52	1.21	Benzo[e]pyrene
72	31.65	1.22	6-amino-1,3-di-2-propenyl-2,4(1H,3H)-Pyrimidinedione
73	31.72	0.79	8.beta.,12.alpha.-Dihydroxysandaracopimar-15-ene-11-one
74	31.94	2.32	2-ethylbutyric Acid, 2,2,2-trifluoroethyl ester
75	32.18	1.64	2-Hydrazino-4,6-dimethylpyrimidine ditms peak 2
76	32.34	1.58	2,3-dihydro-2-hydroxymethyl-5,7-dimethyl-Oxazolo[3,2-E]xanthine
77	32.54	1.43	Hexacosanoic acid, 9-oxo-, methyl ester
78	32.66	1.01	N-BOC-5-iodo-Histamine

79	32.73	1.66	4,6-dimethyl-2-spirocyclohexane-2H-Imidazo[4,5-d] pyrimidine-5,7(4H,6H)-dione
80	33.02	1.27	2-Bromo-myristoyl-glycinamide
81	33.08	0.81	9,19-Cyclolanostan-3-ol, 24,24-epoxymethano-, acetate
82	33.19	0.33	D-Glycero-L-gulo-Octitol, 2,6:5,7-dianhydro-8-deoxy-7-C-methyl-, triacetate
83	33.20	0.30	2- Bromopropionic acid, octadecyl ester
84	33.25	0.29	Imidazole-4-carboxylic acid, 2-fluoro-1-methoxymethyl-, ethyl ester
85	33.30	1.71	Fumaric acid, octadecyl propyl ester
86	33.58	0.48	N-(4-fluorophenyl)-3-[4-(2-hydroxyethyl)-1-piperazi nyl]- Propanamide
87	33.65	0.43	3-Nitrophthalic acid, bis-(2-ethyl-hexyl ester
88	33.71	0.46	Tetradecanal
89	33.76	0.36	2-methyl-4-(1,3,3-trimethyl-7-oxabicyclo[4.1.0]hept-2 -yl)- 3-Buten-2-ol
90	33.98	1.62	2-Methyl-3-[(1S,2S)-1,3,3-trimethyl-2-(2-hydroxyethy l)cyclohexyl]tetrahydrofuran
91	34.09	0.23	1,2,4-Benzenetricarboxylic acid, cyclic 1,2-anhydride, nonyl ester
92	34.16	0.31	Terephthalic acid, butyl 3-methyl-5-methoxypentyl ester
93	34.48	1.56	Dimethylmalonic acid, 2,5-dichlorophenyl undecyl ester
94	34.55	1.05	Dimethylmalonic acid, dodecyl pentafluorophenyl ester
95	34.81	0.59	Isolongifolan-8-ol
96	35.12	1.17	4-ethyl-5-octyl-2,2-bis(trifluoromethyl)- trans-1,3-Dioxolane
97	35.58	0.12	4-methoxy-Cycloheptanone
98	35.74	0.12	Oleic anhydride
99	35.84	0.11	4,4-Bis(dichlorofluoromethyl)-1,2-oxathietane-2,2-di oxide

100	35.91	0.02	.alpha.,.alpha.'-bis(phenoxymethyl)-1,4-Piperazinediethanol
